# Genome Analysis Coupled with Physiological Studies Reveals a Diverse Nitrogen Metabolism in *Methylocystis* sp. Strain SC2

**DOI:** 10.1371/journal.pone.0074767

**Published:** 2013-10-10

**Authors:** Bomba Dam, Somasri Dam, Jochen Blom, Werner Liesack

**Affiliations:** 1 Max Planck Institute for Terrestrial Microbiology, Marburg, Germany; 2 Center for Synthetic Microbiology (SYNMIKRO), Philipps-Universität Marburg, Marburg, Germany; 3 Center for Biotechnology (CeBiTec), Bielefeld University, Bielefeld, Germany; Tel Aviv University, Israel

## Abstract

**Background:**

*Methylocystis* sp. strain SC2 can adapt to a wide range of methane concentrations. This is due to the presence of two isozymes of particulate methane monooxygenase exhibiting different methane oxidation kinetics. To gain insight into the underlying genetic information, its genome was sequenced and found to comprise a 3.77 Mb chromosome and two large plasmids.

**Principal Findings:**

We report important features of the strain SC2 genome. Its sequence is compared with those of seven other methanotroph genomes, comprising members of the *Alphaproteobacteria*, *Gammaproteobacteria*, and *Verrucomicrobia*. While the pan-genome of all eight methanotroph genomes totals 19,358 CDS, only 154 CDS are shared. The number of core genes increased with phylogenetic relatedness: 328 CDS for proteobacterial methanotrophs and 1,853 CDS for the three alphaproteobacterial *Methylocystaceae* members, *Methylocystis* sp. strain SC2 and strain Rockwell, and *Methylosinus trichosporium* OB3b. The comparative study was coupled with physiological experiments to verify that strain SC2 has diverse nitrogen metabolism capabilities. In correspondence to a full complement of 34 genes involved in N_2_ fixation, strain SC2 was found to grow with atmospheric N_2_ as the sole nitrogen source, preferably at low oxygen concentrations. Denitrification-mediated accumulation of 0.7 nmol ^30^N_2_/hr/mg dry weight of cells under anoxic conditions was detected by tracer analysis. N_2_ production is related to the activities of plasmid-borne nitric oxide and nitrous oxide reductases.

**Conclusions/Perspectives:**

Presence of a complete denitrification pathway in strain SC2, including the plasmid-encoded *nosRZDFYX* operon, is unique among known methanotrophs. However, the exact ecophysiological role of this pathway still needs to be elucidated. Detoxification of toxic nitrogen compounds and energy conservation under oxygen-limiting conditions are among the possible roles. Relevant features that may stimulate further research are, for example, absence of CRISPR/Cas systems in strain SC2, high number of iron acquisition systems in strain OB3b, and large number of transposases in strain Rockwell.

## Introduction

In the global methane cycle, aerobic methanotrophic bacteria are the only biological sink for the greenhouse gas methane. They belong to the *Proteobacteria* [1] and *Verrucomicrobia* [2]. The proteobacterial methanotrophs belong to the *Alphaproteobacteria* and *Gammaproteobacteria*. Among them, the alphaproteobacterial members of the genus *Methylocystis* have repeatedly been found to be associated with a wide variety of environments. They have been detected by both cultivation and cultivation-independent molecular techniques in rice paddies [3], [4], different upland and hydromorphic soils [5], [6], [7], landfills [8], [9], peatlands [10], [11], [12], and glacier forefields [13]. These environments are characterized by either oxygen-methane counter-gradients (low-affinity methane oxidation: e.g., rice paddies, peatlands, landfill cover soils) or the consumption of atmospheric methane (high-affinity methane oxidation: e.g., upland soils). Intermediate conditions prevail, for example, in glacier forefields. The ubiquitous distribution of the genus *Methylocystis* may be due to the fact that its members have greater metabolic flexibility than those of other methanotrophic genera. This is in part related to their facultative nature. For example, *Methylocystis* sp. strain H2s can utilize acetate [14] and strain SB2 can utilize acetate and ethanol [15], in addition to methane. *Methylocystis* sp. strain Rockwell has been studied with respect to its ability to utilize different nitrogen sources [16], [17]. Our model organism, *Methylocystis* sp. strain SC2, contains a novel high-affinity particulate methane monooxygenase (pMMO2), in addition to the conventional pMMO1 [18], [19]. The different methane oxidation kinetics of pMMO1 and pMMO2 allow strain SC2 to adapt to a wide range of methane concentrations and thus to changes in its environment [19]. To understand the total genetic potential of this organism, its genome was sequenced [20].

Another major factor determining methanotrophic activity is the source and availability of nitrogen. Diversity of nitrogen metabolism operating in methanotrophs is well known. N_2_ fixation is a well-studied feature among methanotrophs. It has been reported for proteobacterial methanotrophs [21], [22], [23] and the distantly related verrucomicrobial member ‘*Methylacidiphilum fumariolicum*’ SolV [24]. Denitrification is the sequential reduction of nitrate and nitrite to the gaseous compounds nitric oxide (NO), nitrous oxide (N_2_O), and finally N_2_. This process is catalyzed by nitrate, nitrite, nitric oxide, and nitrous oxide reductase, respectively [25]. Incomplete denitrification can lead to the emission of N_2_O, a potent greenhouse gas that contributes to global warming and ozone depletion [26]. Proteobacterial methanotrophs are known to release N_2_O [27], [28], [29], [30], [31], [32]. *Methylococcus capsulatus* Bath and *Methylosinus trichosporium* OB3b have the ability to produce N_2_O from the oxidation of hydroxylamine [33], [34]. Understanding the release and fate of N_2_O is of particular importance for the global nitrogen cycle [35]. Thus, in addition to their methane-oxidizing capabilities, knowledge of their nitrogen metabolism is essential for understanding the ecophysiology of methanotrophic bacteria. Based on a genome-inferred inventory, several key enzymes involved in nitrification and denitrification were suggested to be present in methanotrophs [36]. It was proposed that the oxidation of NH_3_ to nitrite (nitrification) and the production of N-oxides (denitrification) may be interrelated [27], [37], [38]. However, the ability to convert N_2_O to N_2_ has not yet been reported for any of the known methanotrophs.

With the advent of next-generation sequencing technologies, the number of sequenced methanotroph genomes has increased considerably. At present, twelve methanotroph genomes are available in public databases and more are being sequenced. The available sequences include those of the alphaproteobacterial methanotrophs *Methylosinus trichosporium* OB3b [39], *Methylocystis parvus* OBBP [40], *Methylocystis* sp. strain Rockwell [41], *Methylocystis* sp. strain SC2 [20], and the facultative *Methylocella silvestris* BL2 [42]; and the gammaproteobacterial methanotrophs *Methylococcus capsulatus* Bath [43], *Methylomicrobium album* BG8 [44], *Methylomicrobium alcaliphilum* 20Z [45], *Methylomonas methanica* MC09 [46], and the psychrotolerant *Methylobacter tundripaludum* SV96 [47]. In addition, the genome sequences of the acidophilic *Verrucomicrobia* members *Methylacidiphilum infernorum* V4 [48] and ‘*Ma. fumariolicum*’ SolV [49] are available. However, there is no report of any comparative analysis among the methanotroph genomes.

Here, we provide a detailed description of important features of the genome sequence of strain SC2 identified by comparative analysis with the methanotroph genomes available in public databases. In particular, we systematically compared the genome sequence of strain SC2 with those of two other *Methylocystaceae* members, *Methylocystis* sp. strain Rockwell and *Ms. trichosporium* OB3b. Special emphasis was given to genes involved in nitrogen metabolism. Their diverse functional nature in strain SC2 prompted us to perform physiological experiments, in order to verify that this strain is able to fix atmospheric N_2_, produce N_2_O and eventually reduce it to N_2_ by denitrification.

## Results and Discussion

### Genomic analysis of *Methylocystis* sp. strain SC2

#### (a) General features of strain SC2 genome

**Figure 1:**
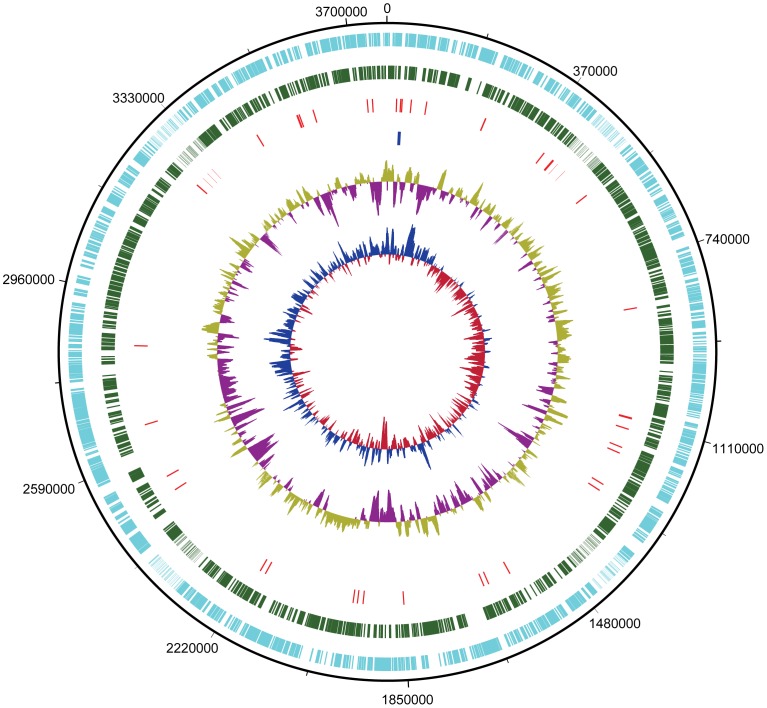
Genome plot of strain SC2. The circles represent from outside to inside: circle 1, DNA base position (bp); circle 2, protein-coding regions transcribed on the plus strand (clockwise); circle 3, protein-coding regions transcribed on the minus strand (anticlockwise); circle 4, tRNA genes; circle 5, rRNA genes; circle 6, G+C content plotted using a 10-kb window (sea green and magenta indicate values greater than and less than the average G+C content, respectively); circle 7, GC skew ([G+C]/[G−C]) plotted using a 10-kb window (blue indicates values above average and red indicates values below average). The genome plot was generated using DNAPlotter version 1.4 from Artemis 12.0, Sanger Institute.

The genome of strain SC2 totals 4,146,594 bp and consists of three replicons: a circular chromosome of 3,773,444 bp (Figure 1) and two plasmids of 229,614 (pBSC2-1) and 143,536 bp (pBSC2-2), with an average GC content of 63, 61 and 60%, respectively [20], [50].

The organization of a genome changes through gene rearrangements. The frequency with which rearrangements occur depends on the activity of mobile and repeated elements such as insertion sequences, transposons, prophage sequences, and plasmids [51]. In strain SC2, we manually identified two putative genomic islands, possibly acquired by transduction. These are defined by a 17-kb region (BN69_1471 to BN69_1495) and a 63-kb region (BN69_1579 to BN69_1669). In both genomic islands, CDS with significant BLAST matches encode phage-related proteins including components of phage head protein, tail protein, integrase, recombinase, and lysozyme. However, most of the genomic island CDS had no significant match in the database. When the chromosome of strain SC2 was scanned for prophage sequences using the widely used software Prophinder [52], no such sequences were detected. This might be due to the fact that the identified islands have lost some phage-related features (like the terminal repeats). The large phage-related island also contains a *hicAB* toxin-antitoxin system (BN69_1608, BN69_1609), which is highly prone to frequent gene rearrangement within a genome and horizontal gene transfer among bacterial and archaeal species [53]. Additional toxin-antitoxin systems encoded on the chromosome include two *mazEF* systems (BN69_0515, BN69_0516; and BN69_2525, BN69_2526) and one *yoeB*–*yefM* system (BN69_3397, BN69_3398). A *relBE* toxin-antitoxin system was identified in the plasmid pBSC2-1 [50]. All toxin-antitoxin systems encode toxins that target diverse cellular functions like DNA replication, mRNA stability, protein synthesis, cell wall biosynthesis, and ATP synthesis [54]. The toxins (RelE, MazF, and YoeB) predicted to be produced in strain SC2 function as site-specific endoribonucleases that cleave mRNA at specific sites and thereby hamper mRNA stability [55], [56], [57], [58]. The HicA toxin, encoded by the *hicAB* system, was proposed to function via RNA cleavage [53]. In normally growing cells, these toxins are coexpressed and neutralized by their cognate antitoxins produced from the second gene of the operon [54]. Presence of multiple toxin-antitoxin systems in the chromosome of strain SC2 might help this bacterium to cope with stress or to undergo programmed cell death under stressed conditions [59], [60].

To identify **C**lustered **R**egularly **I**nterspaced **S**hort **P**alindromic **R**epeats (CRISPRs), the web-based tool “CRISPRFinder” was used [61]. CRISPRs are widespread in prokaryotes. A survey identified them in 83% of 150 archaeal genomes and 46% of 2,356 bacterial genomes analyzed (http://crispr.u-psud.fr/crispr) [62]. CRISPR arrays are composed of highly conserved tandem repeat sequences, varying in size from 23 to 47 base pairs. These repeats are separated by unique ‘spacer’ sequences of similar length, which in most cases have been identified to be of viral origin. CRISPRs are flanked on one side by an AT-rich sequence called the ‘leader’ [62]. CRISPR loci, together with their CRISPR-associated (*cas*) genes, have recently been shown to constitute a defense system that, in bacteria, restricts propagation of intruding viruses and plasmids. CRISPR systems presumably function as transcriptional regulators or RNA-interference-based immune systems [63], [64], [65]. We could not detect any CRISPR-like sequence in the genome of strain SC2. When a similar search was made with the available genome sequences of methanotrophs, CRISPRs ranging from 2 to 6 per genome were identified by “CRISPRFinder” in all of them, except for the genome of *Mce. silvestris* BL2 (Table 1). Likewise, *cas* genes were not found in the genomes of strains SC2 and BL2 but were present in all other methanotroph genomes having CRISPR loci (Table 1). The absence of CRISPRs and *cas* genes might help strain SC2 to maintain and stabilize its two plasmids, as has also been reported for several strains of multidrug-resistant enterococci [66]. Similar to the situation with *Methylocystis* sp. strains SC2 and Rockwell, CRISPRs are absent or present among strains of the same species in lactic acid bacteria [67].

**Table 1:** General features identified in the genomes of the compared methanotrophs.

*Alphaproteobacteria*					*Gammaproteobacteria*			*Verrucomicrobia*
Features	*Methylocystis* sp. strain SC2	*Methylocystis* sp. strain Rockwell	*Ms. trichosporium* OB3b	*Mce. silvestris* BL2	*Mc. capsulatus* Bath	*Mmo. methanica* MC09	*Mm. alcaliphilum* 20Z	*Ma. infernorum* V4
**Accession number**	HE956757	AEVM00000000	ADVE00000000	CP001280	AE017282	CP002738	FO082060	CP000975
**Status**	Complete	149 contigs	173 contigs	Complete	Complete	Complete	Complete	Complete
**Genome size (Mb)**	3.77	4.6	4.9	4.3	3.3	5.05	4.67	2.2
**G+C content (%)**	63	63	66	63	64	51	49	45
**Total no. of CDS**	3,666	4,637	4,472^1^	4,016	3,120	4,494	4,083	2,473
**rRNA operons**	1	1	1	2	2	1	3	1
**No. of tRNA genes**	All (47)	All (51)	All	All	All (46)	All	All (44)	All (46)
**tRNA genes in ** ***rrn*** ** operons** 2	Ile-Ala	Ile-Ala^3^	Ile-Ala^4^	Ile-Ala (in both)	Ile-Ala (in both)	Ile-Ala	Ile-Ala (in all three)	Ala-Ile
***pmoCAB1*** ** operon**	2	1	1	Absent	2	1	1	3
***pmoCAB2*** ** operon**	1	Absent	Absent	Absent	Absent	Absent	Absent	Absent
**Monocistronic ** ***pmoC***	3 (1 in plasmid)	4	1	Absent	1	Absent	-	1
**sMMO-encoding operon**	Absent	Absent	1	1	1	1	-	Absent
***pxmABC*** ** operon**	Absent	Absent	Absent	Absent	Absent	Absent	-	Absent
**Serine pathway genes**	Present	Present	Present	Present	Incomplete	Absent	Absent	Present (partial)
**RuMP pathway genes**	Absent	Absent	Absent	Absent	Present	Present	Present	Absent
**Plasmid(s)**	2^5^	NR^6^	NR	NR	NR	NR	1^7^	NR
**CRISPRs** 8	0	2	2	0	2	4	3	4
**No. of ** ***cas*** ** genes** 9	0	2	5	0	10	7	18	8

1 The number of CDS predicted in the genome announcement is 4,503, while the submitted sequence actually contains 4,472 CDS.

2 tRNA genes were identified in 16S-23S spacer region of the rRNA operons.

3 rRNA operon present in contig 219 (AEVM01000005).

4 rRNA operon present in contig 00159 (NZ_ADVE01000118).

5 Size of plasmids: pBSC2-1 (FO000001), 223 kb; and pBSC2-2 (FO000002), 143 kb.

6 NR – not reported.

7 Size of plasmid: MEALZ_p (FO082061), 128 kb.

8 Abbreviation: **C**lustered **R**egularly **I**nterspaced **S**hort **P**alindromic **R**epeats; identified using the online tool “CRISPR finder”.

9 CRISPR-associated (*cas*) genes were predicted using the RAST server.

#### (b) CDS involved in replication, transcription, and translation

**Figure 2:**
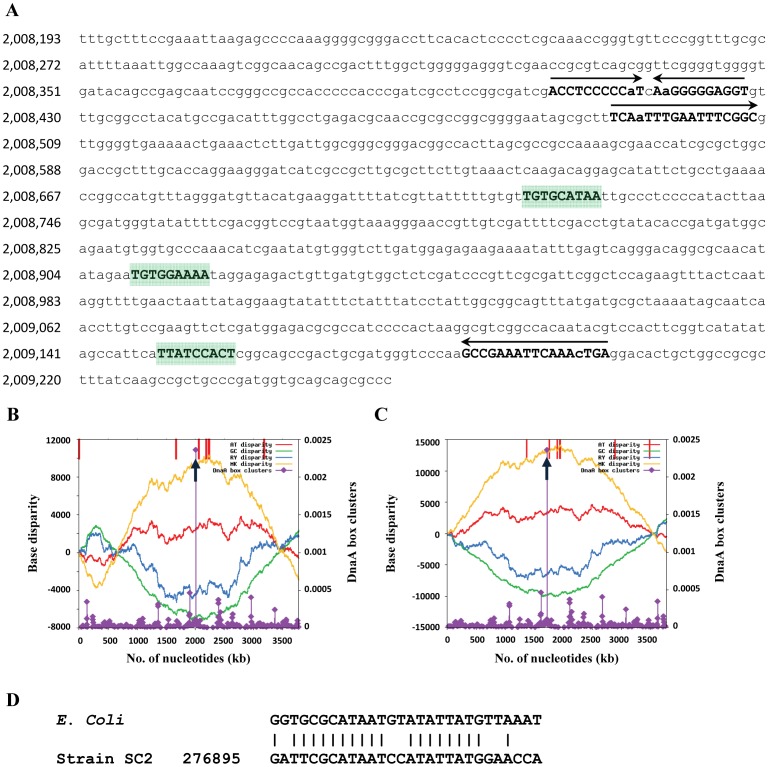
Prediction of the *oriC* region by Ori-Finder. (A) 1,063-bp sequence (2,008,193 bp to 2,009,255 bp) of the predicted *oriC* site. Three *dnaA* box motifs identified using the *Escherichia coli* specific *dnaA* boxes are bold-faced and highlighted. Palindromic repeats identified in this region are marked by arrows at the top. (B, C) The Z-curves measuring the disparity between the percent content of AT (red lines), GC (green lines), RY (blue lines) and MK (yellow lines) for the original sequence (B) and the rotated sequence (C). It should be noted that the coordinate origin of the rotated sequence begins and ends in the maximum of the GC disparity curve. Short vertical red lines at the top show the locations of indicator genes, such as *dnaA*, *dnaN*, *gidA*, and *hemE*. The upward black arrow indicates the position of the predicted *oriC*. Purple peaks with diamonds indicate DnaA box clusters. (D) Pairwise alignment between the *dif* sites located in the genomes of *E. coli* and strain SC2. In strain SC2, the *dif*-like sequence is located from nucleotide position 276,895 to 276,922 (almost halfway of the deduced *oriC*) and matches at 20 nucleotide positions with the 28-bp *dif* sequence of *E. coli*.

The analysis of GC skewing (Figure 1) did not reveal a clear inversion pattern in the chromosome. Therefore, it was not possible to determine the origin of replication (*oriC*) by this approach. However, we could identify the putative *oriC* region using the Ori-Finder program [68], with parameters adjusted to specific DNA boxes of *E. coli* and one unmatched site permitted. This tool makes predictions based on the following features: (i) compositional strand asymmetry (estimated using the Z-curve program), (ii) distribution of DnaA boxes (either of the *Escherichia coli* type or species-specific), (iii) location of indicator genes (such as *dnaA*, *hemE*, *gidA*, *dnaN*, *hemB*, *maf*, *repC*, etc.), and (iv) phylogenetic relationships [68], [69]. The putative *oriC* was identified within a 1063-bp region (2,008,193 bp to 2,009,255 bp). Its GC content is 53%, which is 10% lower than the GC content of the chromosome as a whole (Figure 2). Three *dnaA* box motifs could be identified within this region using the *E. coli*-specific *dnaA* box sequence as the reference. Two palindromic repeats were also identified in this region (Figure 2A). The predicted *oriC* is not located in the vicinity of any of the three DnaA-encoding CDS (BN69_0001, BN69_3094, BN69_3291). The Ori-Finder program does not consider *dif* sites while making *oriC* predictions [68], [69]. However, we could detect a *dif* site (276,895 bp to 276,922 bp) located almost halfway of the predicted *oriC* (Figure 2D). The *dif* site has been shown to be associated with the termination region of bacterial chromosomes [70] and acts as the recognition site for the XerCD proteins [71]. These are involved in postreplication recombination events. CDS encoding XerCD proteins (BN69_2958 and BN69_2761, respectively) were identified in the chromosome. The sum of these findings provides evidence for the correct prediction of *oriC*. Nevertheless, experimental validation is needed to unambiguously locate *oriC*.

Twenty-four CDS encode proteins whose products are involved in transcription. Among these are the following: Two transcriptional elongation factors (GreA [BN69_0089] and GreB [BN69_2117]), three transcriptional antitermination factors (NusA [BN69_2506], NusB [BN69_0468], and NusG [BN69_1633]), and one transcriptional termination factor rho (BN69_2165). CDS encoding α, β, β′ and ω subunits (BN69_1255, BN69_2895, BN69_2894, and BN69_1074, respectively) of bacterial DNA-directed RNA polymerase were also detected. Nine CDS are devoted to the synthesis and maintenance of the RNA polymerase sigma factor.

Several components of the translation system were identified, including a single copy of the 16S-23S-5S ribosomal RNA operon. The 16S and 23S rRNA genes are interspersed by two transfer RNA (tRNA) genes for isoleucine and alanine. This arrangement is commonly found in proteobacterial rRNA operons [72], [73] and more frequently among members of the *Alphaproteobacteria* [72]. A similar organization of tRNA genes within the rRNA operon was observed in all the methanotroph genomes examined, except for the *Verrucomicrobia* member where the arrangement is in the reverse order, Ala-Ile (Table 1). The full complement of 54 ribosomal proteins required for ribosome biosynthesis was identified in the chromosome. This includes 21 and 33 CDS, respectively, encoding components of the small and large ribosomal subunits. In total, 47 tRNA genes covering 20 amino acids were identified. No tRNA for the translation of the amino acid selenocysteine (tRNA-Sec) was found, corroborating that the number of bacteria with tRNA-Sec is much less than previously expected [74]. Other important CDS of the translation system include aminoacyl-tRNA synthetases, responsible for precise attachment of all 20 amino acids to their cognate transfer RNAs. Three bacterial initiation factors (IF-1 [BN69_0875], IF-2 [BN69_2508], and IF-3 [BN69_0601]) and three peptide release factors (RF-1 [BN69_0797], RF-2 [BN69_2418], and RF-3 [BN69_0479]) were identified. The latter are responsible for the recognition of the stop codons UAA, UAG, and UGA to terminate translation.

#### (c) CDS involved in methanotrophic mode of life

The chromosome of strain SC2 contains all the genes required for a methanotrophic lifestyle, including two copies of the conventional *pmoCAB1* operon and a single copy of the novel *pmoCAB2* operon (Table S1). In addition, three monocistronic *pmoC* paralogs were identified, with one present on plasmid pBSC2-2 [50]. As expected for an alphaproteobacterial methanotroph, we could identify the genes involved in the serine pathway of formaldehyde assimilation, but not those involved in the RuMP pathway.

The monocistronic *pmoC1_Gs_* (BN69_0852) is identical to the homolog present in the *pmoCAB1* operons. Interestingly, the CDS (BN69_0853) present directly upstream of this monocistronic gene encodes an ATP-dependent zinc metalloprotease, FtsH1 protein. No such gene is present in the vicinity of *pmoC2_Gs_*. When we searched the genome of strain Rockwell, *ftsH* genes were found immediately downstream of two of its four monocistronic *pmoC* genes (ZP_08074599 and ZP_08075129). Characterized in *Escherichia coli*, FtsH is a membrane-bound ATP-dependent protease that is involved in the degradation of uncomplexed or misfolded integral membrane proteins and short-lived cytoplasmic proteins [75], [76], [77]. FtsH functions as a protein-filtering system and ensures that only correctly folded protein is incorporated into the membrane. Based on the presence of *ftsH* in close association to monocistronic *pmoC*, whose exact function is yet to be identified, one may speculate that this monocistronic gene (along with FtsH) might act as a sensor to screen whether properly folded pMMO is incorporated into the membrane of these methanotrophs. However, this needs to be experimentally verified, before claiming an exact function in the two strains, SC2 and Rockwell. No *ftsH* gene was detected in the vicinity of the monocistronic *pmoC* in strain OB3b (EFH02634) and *Mc. capsulatus* Bath (YP_112829). A possible explanation for the absence of this gene might be the additional presence of the soluble form of methane monooxygenase (sMMO) in these bacteria.

#### (d) Nitrogen metabolism-related genes

Genome sequence analysis revealed the presence of a large number of genes whose products are presumably involved in nitrogen metabolism. This includes N_2_ fixation, ammonium transport, assimilatory nitrate/nitrite reduction, hydroxylamine detoxification, and denitrification (Table S1) [20]. A full chromosome-encoded complement of N_2_ fixation-related genes (34 CDS) was identified. The genes mostly clustered together, suggesting that strain SC2 is capable of utilizing N_2_ as a nitrogen source (see below).

The first step in nitrification is the oxidation of ammonia to hydroxylamine. Ammonia monooxygenase (AMO) performs this step in ammonia-oxidizing bacteria. AMO and pMMO are known to be homologous. They are encoded by three contiguous genes that are organized in the order *amoCAB/pmoCAB* [78], [79]. Due to their structural homology, pMMO can also oxidize ammonia [80]. Hydroxylamine is highly toxic and bacteria that can oxidize ammonia must have effective mechanisms to detoxify it. All ammonia oxidizers and some methanotrophs are known to use hydroxylamine oxidoreductase (HAO) to oxidize hydroxylamine to nitrite. However, the difference lies in the fact that ammonia oxidizers, but not methanotrophs, use this step for energy production [27]. The *haoAB* operon, encoding this enzyme, was identified in the chromosome of strain SC2 (BN69_3242, BN69_3241). In addition, the chromosome encodes a hydroxylamine reductase or hybrid cluster protein (BN69_0431) that presumably detoxifies hydroxylamine by reducing it to ammonia. A second copy of hydroxylamine reductase was identified in pBSC2-2 [50]. Thus, strain SC2 apparently possesses two different systems to detoxify hydroxylamine. None of the other genome-sequenced methanotrophs are known to possess both detoxification systems.

Methanotrophs are reported to produce N_2_O during ammonia oxidation [29], [30], [32]. The chromosome does not encode any enzyme that can contribute to this function in strain SC2. However, a CDS encoding nitric oxide reductase (homolog of *norB*) is present in each of the two plasmids [50]. And most interestingly, a complete nitrous oxide reductase operon (*nosRZDFYX*) was identified in pBSC2-2 [50]. The cluster contains the key functional gene *nosZ*. In addition, it includes *nosR* and *nosX*. The two genes are exclusively present in typical *nos* clusters of denitrifiers as, for example, in *Bradyrhizobium japonicum* strain USDA 110 [81]. However, the exact origin of this plasmid-borne *nos* operon could not be predicted as BLAST searches of its individual genes showed homologs from diverse origin (Table S2).

Two genes were predicted to encode ammonium transporters (BN69_0915 and BN69_0931), suggesting that ammonia is an important nitrogen source for strain SC2. We also identified genes encoding the high-affinity ATP-driven potassium transporter (*kdpABC*). These three genes encoding the potassium transporter ATPase (BN69_2487 to BN69_2489) are located immediately downstream of an osmosensitive signal transduction histidine kinase (*kdpD*, BN69_2486) and a two-component transcriptional regulator (*kdpE*, BN69_2485). The potassium transporter has been shown to also transport ammonium ions. This is due to the similarity between ammonium and potassium ions, both in terms of charge and size [82]. Thus, this transporter may facilitate transport of ammonium ions in strain SC2. The chromosome includes a full complement of genes (BN69_2468 to BN69_2473) for transport of nitrate/nitrite across the cytoplasmic membrane and their reduction to ammonia. This is referred as the assimilatory nitrate/nitrite reductase system (Nas). However, genes encoding the Nar or Nap type of nitrate reductase were not found.

Ammonium is the most reduced form of inorganic nitrogen prior to its incorporation into organic nitrogen compounds via glutamate or glutamine, which serve as the key nitrogen donors for biosynthetic processes. The incorporation can occur via the glutamine synthetase/glutamate synthetase (GS) or NADPH-dependent glutamine oxoglutarate amidotransferase (GOGAT) pathway, or the glutamate dehydrogenase (GDH) pathway [83]. In bacteria, GS and GOGAT function as alternative pathways of ammonia assimilation and operate when ammonia is present in the growth medium at low levels [84]. The SC2 chromosome encodes GS (BN69_0652) and both the large (BN69_3582) and small (BN69_3584) subunits of GOGAT. GDH (BN69_0999) was also identified.

In addition to enzymes of the nitrogen metabolism, many potential regulatory components involved in this process are encoded by the chromosome, including sigma factor RpoN (BN69_2202). This factor is essential for the expression of several nitrogen regulons, such as the *ntr* (nitrogen regulation) and *nif* (N_2_ fixation) operons. However, RpoN is not only involved in the nitrogen metabolism, but also controls the regulation of a number of other metabolic processes in bacteria. For example, in *Pseudomonas putida*, RpoN was found to be involved in processes like motility and expression of plasmid-encoded catabolite operons, and in determining the ability of *P. putida* to utilize diverse nitrogen and carbon sources [85].

The genes encoding the nitrogen signaling cascade (*ntrBC* [BN69_0222, BN69_0223] and *ntrYX* [BN69_0224, BN69_0225]) and a gene for uridyltransferase (*glnD* [BN69_3100]) were also identified. NtrB and NtrC act as a two-component signal transduction cascade for nitrogen regulation, where NtrB is the bifunctional histidine kinase and NtrC is its cognate response regulator [86]. They are required for maximal GS synthesis. The second transcription regulator, *ntrYX*, is located immediately upstream to *ntrBC*. The NtrY and NtrX proteins constitute a two-component regulatory system that is involved in N_2_ fixation and metabolism [87]. All *ntr* genes are clustered together in an operon, *nifR3*-*ntrB*-*ntrC*-*ntrY*-*ntrX*. The fifth component of this operon, *nifR3* (BN69_0221), encodes a tRNA-dihydrouridine.

The identification of a full complement of N_2_ fixation-related genes and plasmid-borne genes for denitrification prompted us to test strain SC2 for these metabolic capabilities.

### Physiological studies on the nitrogen metabolism of strain SC2

#### (a) N_2_ fixation

**Figure 3:**
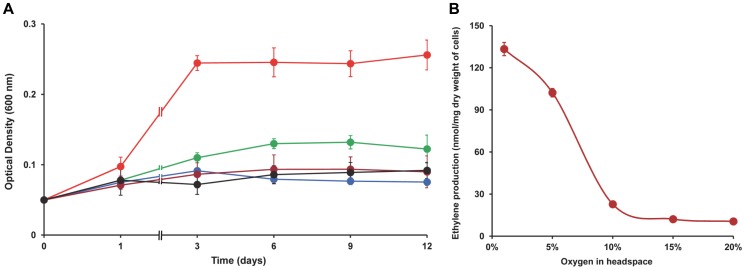
N_2_ fixation by strain SC2. (A) Growth dynamics (OD_600_) of strain SC2 in batch cultures on N-free medium (with atmospheric N_2_ as sole nitrogen source). Oxygen concentrations of 1% (blue), 5% (green), 10% (red), 15% (brown) and 20% (black) were used to test their effect on N_2_ fixation-mediated growth. Note that the x-axis is not in scale. (B) Effect of oxygen on the nitrogenase activity (acetylene reduction assay) in strain SC2. Ethylene production was measured after 24 hours of incubation under different concentrations of oxygen in the headspace. Data points are means ±SD of three separate experiments.

The ability of strain SC2 cells to fix N_2_ was tested in nitrogen-free mineral salts medium. The serum bottles were flushed with N_2_ followed by the addition of methane (20%). Different initial concentrations of oxygen were tested. Maximum growth was observed with 10% oxygen in the headspace, while 5% oxygen allowed little growth (Figure 3A). Insignificant increase in OD_600_ value was observed under lower (1%) and higher (15% and 20%) oxygen concentrations. Most likely, the optimal concentration for growth of strain SC2 under N_2_-fixing conditions is between 5% and 10% oxygen. In respect to their N_2_-fixing activities, methanotrophic bacteria are known to vary in oxygen sensitivity. In batch cultivation, the requirement of low oxygen concentration has been demonstrated for *Methylobacter luteus* (<2%), ‘*Ma. fumariolicum*’ SolV (<2%), *Methylocystis* sp. strain T-1 (<6%) and *Mc. capsulatus* Bath (<10%) [21], [22], [24], [88]. In contrast, some other methanotrophs are able to fix N_2_ at higher oxygen concentrations including, for example, *Ms. trichosporium* OB3b (15–17%) and *Methylocapsa acidiphila* B2T (atmospheric oxygen concentration) [22], [23], [89].

The cells growing in N-free medium were tested for nitrogenase activity using the acetylene reduction assay. As methane oxidation or, more precisely, the activity of methane monooxygenase is known to be inhibited by acetylene [90], [91], methanol was used as a source of energy and reducing power in the assay [91]. Ethylene production was detected after 3 hours of incubation with acetylene, and the produced amount increased linearly for more than 24 hours. The 3-hour lag prior to ethylene production was also observed for other methanotrophs, such as *Mc. capsulatus* Bath and ‘*Ma. fumariolicum*’ SolV [24], [92]. Ethylene production measured after 24 hours of incubation was found to be affected by oxygen concentration in the headspace, with highest amount produced at 1% oxygen (133 nmol ethylene/mg dry weight of cells). The amount of ethylene produced decreased with increasing concentration of oxygen (Figure 3B). Thus, acetylene reduction activity was affected by the oxygen concentration as also observed in other aerobic diazotrophs including methanotrophs [21]. In principle, both the growth experiments and the nitrogenase activity assays consistently showed the detrimental effect of increasing oxygen concentration to N_2_ fixation. However, while growth yield was highest at 10% oxygen, nitrogenase activity was greatest at around 1% oxygen in the headspace.

#### (b) Denitrification

**Figure 4:**
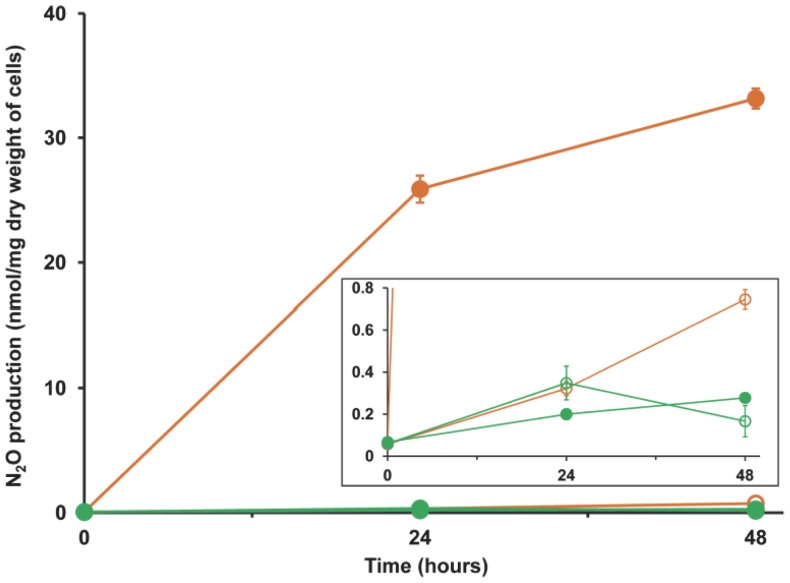
N_2_O production by strain SC2. Cells were incubated in NMS, either in the presence (filled symbol) or absence (open symbol) of 10% acetylene. Assays were performed both under anaerobic (orange) and aerobic (green) conditions. Data points are means ±SD of three separate experiments. The inset shows the same graph with a y-axis zoomed in for the range 0 to 0.8.

Under standard growth conditions, strain SC2 was found to accumulate only a negligible amount of N_2_O, both under aerobic as well as anaerobic conditions (Figure 4). One explanation for this result may be the presence of a non-functional (less-active) nitric oxide reductase. Another possibility may be the presence of an active/functional nitrous oxide reductase produced from the plasmid-borne *nos* operon, thereby resulting in the rapid conversion of N_2_O to N_2_. To examine the second possibility, we checked N_2_O production after blocking the *nos* activity with purified acetylene [93], [94], [95]. As acetylene is also a potent inhibitor of methane monooxygenase [90], we performed this experiment either by adding methanol or without a carbon source. Under aerobic conditions, N_2_O production was negligible even after acetylene addition. This was expected as denitrification is an anaerobic process. However, under anaerobic conditions, acetylene inhibition was pronounced and N_2_O accumulated in the headspace. After 48 hours of incubation, the methanol-fed cells produced 33 nM N_2_O per mg dry weight of cells (Figure 4). Nearly equal amount of N_2_O (28 nM per mg dry weight of cells) was produced when cells were incubated under starved condition (data not shown). In the methanol-fed cultures, methanol could act as an alternative electron donor in the absence of methane. However, the ability of the cells to produce N_2_O under starved conditions needs further experimental investigation. A possible explanation might be that strain SC2 cells are able to use intracellular poly-beta-hydroxybutyrate (PHB) as a source of carbon during starvation periods. PHB was found to be produced by almost all alphaproteobacterial methanotrophs. Actually, strain SC2 produced the maximum amount of PHB among five different *Methylocystis* strains and the third highest among all alphaproteobacterial methanotrophs tested [96]. During sequence analysis, genes encoding PHB metabolism-related enzymes were detected in the chromosome of strain SC2. These include two PHB depolymerases (BN69_2992 and BN69_3262), one polyhydroxyalkonate synthesis repressor (PhbR [BN69_3069]), an acetyl-CoA acetyltransferase (PhbA [BN69_3068]), one acetoacetyl-CoA reductase (PhbB [BN69_3067]), and two phasin homologs (BN69_0212, BN69_1107). The *phbR*, *phbA*, and *phbB* genes form a cluster, but in an orientation different from that in the PHB-producing methanotroph *Methylocystis parvus* OBB (*phbABR*) [40]. The use of PHB as a reducing power for denitrification has been shown in microbial granules in bioreactors [97]. Moreover, strains of *Methylocystis parvus* were reported to be able to ferment intracellular PHB and use it as a reserve energy source under anoxic conditions [98], [99]. These overall findings support the possibility that PHB degradation and denitrification are interlinked in strain SC2.

**Figure 5:**
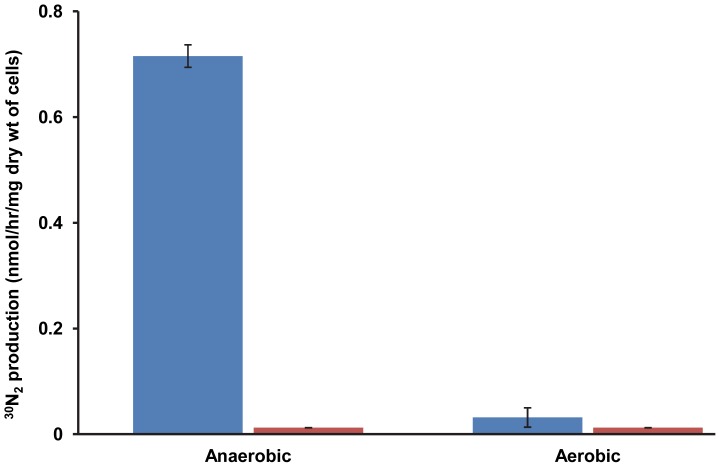
Denitrification-mediated N_2_ production by strain SC2. ^30^N_2_ production was measured after fifteen days for cells incubated in NMS containing either K^15^NO_3_ (blue) or KNO_3_ (orange). The assays were performed under both anaerobic and aerobic conditions. Data points are means ±SD of three separate experiments.

To ultimately prove the emission of N_2_ and thus the operation of a plasmid-encoded denitrification process in strain SC2, a tracer experiment was performed using ^15^N-nitrate (K^15^NO_3_) as the sole nitrogen source. Under anoxic conditions, we could detect accumulation of about 0.7 nmol ^30^N_2_/hr/mg dry weight of cells (Figure 5). Taking all these findings together, strong evidence is provided that strain SC2 possesses a complete denitrification pathway. However, its exact ecophysiological role still needs to be elucidated. Detoxification of toxic nitrogen compounds and energy conservation under oxygen-limiting conditions are among the possible roles.

### Comparative genomics

#### (a) Comparative analysis of methanotroph genomes

Comparative genomics is commonly used for the study of closely related strains of a single species, species of a particular genus, or species of related genera [100]. However, members of broader taxonomic ranks have also been compared, like those belonging to the same family, such as *Pseudonocardiaceae* [101] and *Methylophilaceae* [102], or to different families [103]. Here, we compared the genome sequences of eight methanotrophs belonging to different classes and phyla. These include the genomes of four alphaproteobacterial and three gammaproteobacterial methanotrophs, and one from the recently described methanotroph of the phylum *Verrucomicrobia*. The remaining four publicly available genome sequences were not included in the comparative analysis. This includes the genome of the second verrucomicrobial methanotroph, ‘*Ma. fumariolicum*’ SolV, which is available in draft form; and three proteobacterial members, *Methylocystis parvus* OBBP, *Mm. album* BG8 and *Mb. tundripaludum*, for which no genome annotations were available. The main features identified in the compared genomes are summarized in Table 1. The genome sequences of strains Rockwell and OB3b are available in draft form and consist of numerous contigs. As strain SC2 is their closest relative, its finished genome sequence was used as the reference for assembling their contigs. The chromosome and the two plasmids of strain SC2 were concatenated to a single sequence containing 4,049 CDS, collectively referred to as the genome. The chromosome and plasmid sequences of *Mm. alcaliphilum* 20Z were also concatenated into a single file, while the other genomes had no plasmids. The genome sequences were then subjected to comparative analysis, using the EDGAR platform [100].

The pan-genome or the full complement of genes present in the eight methanotroph genomes sums up to 19,358 CDS. On the contrary, the set of genes shared by all eight methanotrophs was represented by only 154 CDS. This core genome represents the conserved genetic backbone and encodes basic cellular machineries, such as DNA replication, DNA repair, transcription, protein biosynthesis, cell division and a few chaperon and heat-shock proteins (Table S3). None of the genes encoded by the plasmids of strain SC2 are included in the core gene set. The number of core genes is remarkably low, presumably due to the fact that the compared methanotrophs are from phylogenetically very distinct groups. However, in the verrucomicrobial genome, 35% of genes were found to be related to proteobacteria [48]. The set of core genes increased to 328 CDS, when this genome was removed from the calculation.

Although the methanotrophs compared in this study exhibit the same basic metabolic capability of utilizing methane as carbon and energy source, none of the key genes involved in the process were shared by all of them. This is due to the fact that methanotrophs have distinct enzyme systems for metabolizing methane. Some possess either pMMO or sMMO, while others have the ability to produce both key enzymes. They use different pathways for assimilation of formaldehyde into cell biomass. While alphaproteobacterial methanotrophs use the serine pathway, gammaproteobacterial methanotrophs employ the RuMP pathway. Similar to our findings, a very small set of core genes was observed between five genera of the family *Methylophilaceae* [102]. Most interestingly, although the central metabolism in all compared *Methylophilaceae* members was methylotrophy, their core genome was devoid of genes encoding some of the *bona fide* methylotrophy-related functions, such as methanol dehydrogenase, methylamine dehydrogenase, and the H_4_MPT-linked formaldehyde oxidation [102].

**Figure 6:**
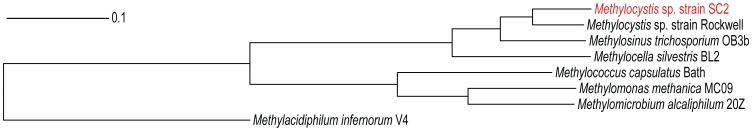
Neighbor-joining tree constructed for the methanotrophic core genome. The tree is based on the alignment of 154 CDS that are common to all eight methanotroph genomes used for comparative analysis. Non-matching parts of the alignments were eliminated prior to tree construction. The individual gene alignments were combined into one concatenated alignment. The neighbor-joining tree was constructed using EDGAR. All branches of the phylogenetic tree showed 100% bootstrap support based on 500 replications. See ‘Materials and Methods’ for further details.

A phylogenetic analysis was performed using the concatenated multiple alignments of all 154 core genes (Table S3) and the neighbor-joining method for tree construction. In the core genome tree, members of the proteobacterial methanotrophs were grouped into two distinct clades, with the distantly related genome of *Ma. infernorum* V4 forming the outgroup (Figure 6). This clustering agreed well with the known phylogeny of methanotrophs as inferred from the comparative analysis of 16S rRNA and *pmoA* gene sequences [2]. To identify the core gene content that is specific to the genomes of the alphaproteobacterial or gammaproteobacterial methanotrophs, both groups were analyzed separately. While the four alphaproteobacterial methanotrophs shared 1,306 CDS, the three gammaproteobacterial methanotrophs shared 1,193 CDS among themselves (Figure S1).

#### (b) Comparative analysis among *Methylocystaceae*

**Figure 7:**
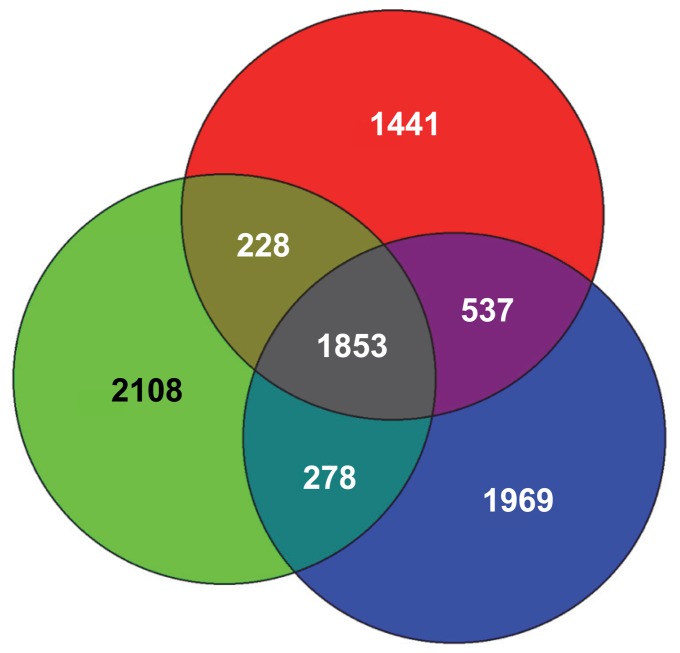
Venn diagram showing the number of CDS unique to and shared by the *Methylocystaceae* members. Data analysis was performed using the genomes of strain SC2 (red), strain Rockwell (blue) and *Ms. trichosporium* OB3b (green). Numbers in circles indicate the number of unique CDS, while those in intersections represent the number of orthologous CDS common to two or more strains. Orthologs were detected by reciprocal best BLASTP matches with the EDGAR software.

**Figure 8:**
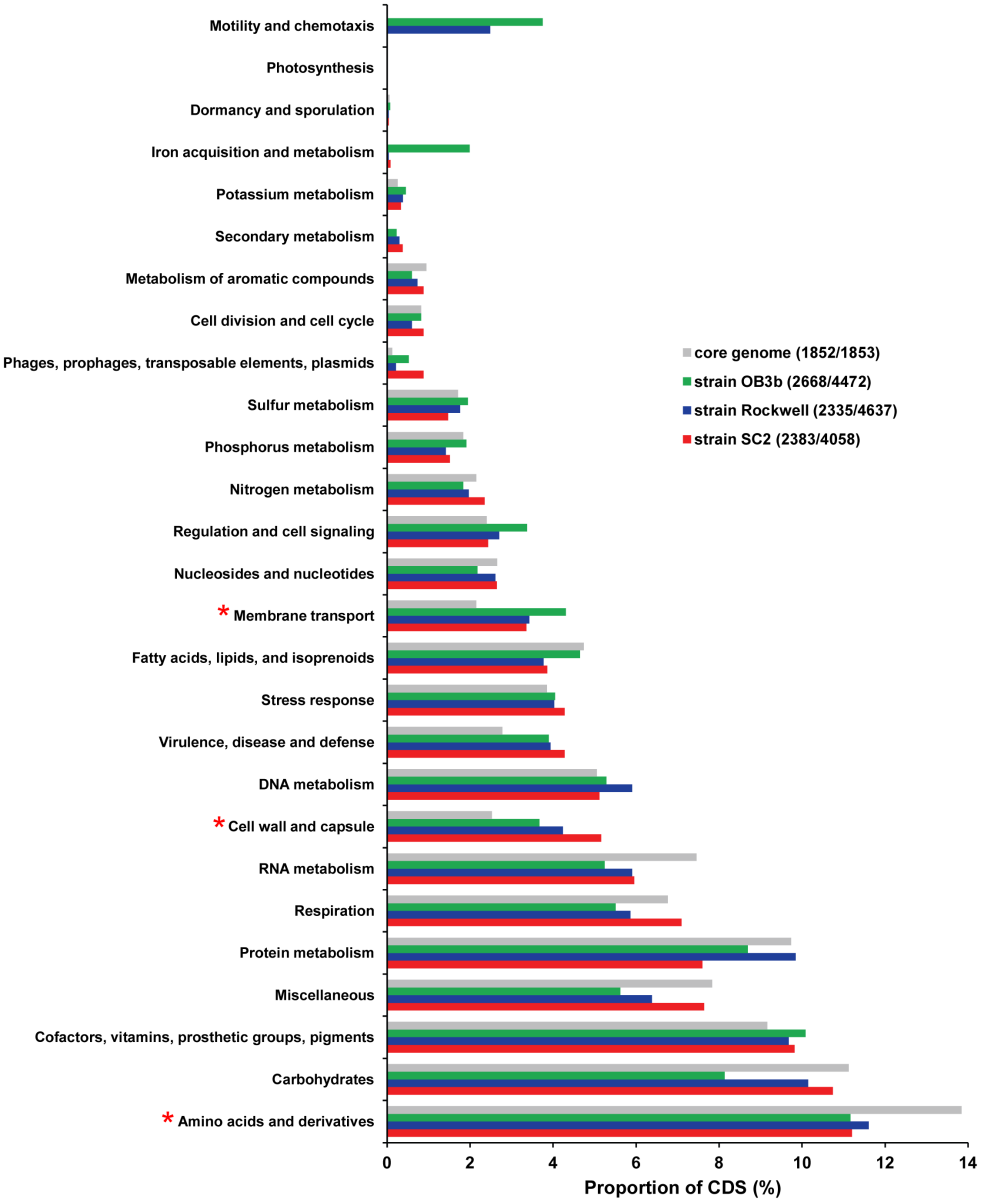
Functional classification of genes identified in members of the *Methylocystaceae*. The gene content of strain SC2 (red), strain Rockwell (blue) and *Ms. trichosporium* OB3b (green) and that of the core genome shared by them (grey) was subjected to functional classification by the RAST server. CDS were classified into 27 functional categories using the SEED subsystem. Numbers in parentheses next to the strain names indicate the number of CDS assigned to the SEED subsystem out of the total number of CDS present in the particular genome. The proportion of CDS (x-axis) assigned to a particular subsystem was calculated by dividing the number of CDS assigned to this category by the total number of CDS assigned to the SEED subsystem database. The functional categories were arranged according to the number of CDS assigned for strain SC2 to each category. The number of CDS classified for the individual strains and their core genome into each SEED subsystem was subjected to statistical analysis using STAMP. A *p*-value cutoff of 0.05 was used to determine significant differences. Subsystems showing significant differences are marked by an asterisk.

Three of the eight methanotroph genomes that were comparatively analyzed belong to the family *Methylocystaceae* in the *Alphaproteobacteria*. In addition to strain SC2, this includes *Methylocystis* sp. strain Rockwell and *Ms. trichosporium* OB3b. Their 16S rRNA gene sequences show a high similarity of respectively 99% and 96% to that of strain SC2. To get an insight into the genomic variation among these three alphaproteobacterial methanotrophs, their genomes were compared in greater detail. Their pan-genome totals 8,374 CDS, while they shared a set of 1,853 CDS among themselves (Figure 7). The predicted products of these common genes are distributed across almost all functional categories of the SEED subsystems (Figure 8). The number of genes assigned to three subsystems, namely, ‘cell wall and capsule’, ‘membrane transport’ and ‘amino acid and derivatives’, showed significant differences between the individual strains and their core genome (Figures 8, S2). Apart from the conserved core genome of all methanotrophs mentioned above, this includes additional genes involved in basic cellular functions. In addition, they also share genes involved in maintaining a methanotrophic lifestyle. These encode, among other proteins, pMMO, methanol dehydrogenase, pyrroloquinoline quinone cofactor biosynthesis proteins, and formate dehydrogenase. Thirty-four nitrogen metabolism-related genes are also shared. These include genes related to N_2_ fixation, ammonia assimilation, and assimilatory nitrate/nitrite reduction. In addition to the core genes, strains SC2 and Rockwell share, respectively, 228 and 278 genes with strain OB3b, while they share 537 genes among themselves (Figure 7). Genes that need to be specifically mentioned include the different hydroxylamine detoxification systems shared by strain SC2 with either strain Rockwell (*haoAB*) or strain OB3b (*hcp*). Eleven percent of the genes shared between strains Rockwell and OB3b are involved in flagella biosynthesis. Although the motility of *Ms. trichosporium* OB3b is well known [104], all *Methylocystis* spp. studied so far, including strain SC2, are reported to be non-motile [1], [105], [106], [107], [108], [109]. This is due to the absence of flagella and, as expected, no flagella biosynthesis-related genes were detected in strain SC2 (Figure 8). However, although no published evidence is available for the motility of strain Rockwell, presence of genes responsible for flagella biosynthesis may suggest that this strain is motile. Another interesting finding is that 53 CDS categorized in the subsystem ‘iron acquisition and metabolism’ are present only in the genome of strain OB3b. These include genes involved in iron acquisition and siderophore production (Figure S2). In contrast, strains Rockwell and SC2, respectively, contain only one and two of these genes. The ability of strain OB3b to produce siderophores, albeit in small amounts, was previously shown, using the Fe-chrome azurol S (CAS) plate assay [110].

The three *Methylocystaceae* members shared approximately half of their CDS, while the other half is unique to the respective strain. Presence of a large number of unique genes even among closely related genomes has been frequently observed [100]. Interestingly, the majority of the genes unique to the individual strain are novel or conserved hypothetical. Only a small proportion could be assigned to functional groups in the SEED subsystem, using the RAST server for analysis. This includes 176 (out of 1,441), 100 (out of 1,969) and 302 (out of 2,108) CDS present in the genomes of strains SC2, Rockwell and OB3b, respectively. Among the enzymes encoded by the 1,441 unique genes identified in strain SC2, those that need special mention are the high-affinity pMMO2 and the plasmid-encoded nitric oxide and nitrous oxide reductases. In addition, strain SC2 possesses two *pmoCAB1* operons, while strains Rockwell and OB3b harbor a single copy of *pmoCAB*. Based on manual search, we could identify a large number of unique genes (173) in strain Rockwell to encode different families of transposases. The number of transposase-encoding genes was quite low in the unique gene set of strains SC2 (45) and OB3b (25). Five such genes were found to be shared by strains Rockwell, SC2, and OB3b. In fact, the number of transposases encoded by the genome of strain Rockwell was almost five times more than the average number of such genes (∼38) detected in 2,137 complete genome sequences analyzed [111]. This may suggest that genome rearrangements occur more frequently in strain Rockwell than in the other two *Methylocystaceae* members. The unique genes present in strain OB3b are those encoding for the soluble methane monooxygenase, iron acquisition systems, urea decomposition system, a large number of membrane transporters and systems imparting resistance to antibiotics and toxic compounds.

### Final remarks

Annotation and comparative analysis of the genome sequence of strain SC2 provide detailed insights into the lifestyle and metabolic potential of this bacterium. Genome analysis coupled with physiological experiments confirmed that strain SC2 possesses diverse nitrogen metabolism-related pathways. This includes the capability to fix atmospheric N_2_ and perform a complete denitrification process, suggesting that strain SC2 is able to thrive under oxygen- and nitrogen-limiting conditions. Its capability to survive in low-methane environments has already previously been shown. The functionality of the plasmid-encoded nitrous oxide reductase is unique to known methanotrophs. Under the tested conditions, the enzyme efficiently converts N_2_O to N_2_. The presence of the complete *nos* operon and a monocistronic *pmoC* on pBSC2-2 suggests that at least this plasmid confers important metabolic traits to strain SC2. Absence of CRISPR/Cas systems may have allowed strain SC2 to acquire and maintain its two plasmids. Comparative genomics across the major methanotroph groups revealed that, although performing the same key metabolic processes, they have very few genes in common. However, the three *Methylocystaceae* members share almost half of their genes. These encode (among other) the central metabolic pathways for methane oxidation and nitrogen fixation. On the other hand, they clearly differ in their genetic potential. This includes the presence of the high-affinity pMMO2 and plasmid-encoded nitrous oxide reductase in strain SC2, high number of iron acquisition systems in strain OB3b, and motility-related genes and predicted genome instability in strain Rockwell (the latter derived from the large number of transposase genes).

## Materials and Methods

### Growth conditions

Strain SC2 was cultivated in nitrogen-containing (1 g KNO_3_ per litre) mineral salts medium (NMS) without any vitamin supplement [112]. In N-free medium, no nitrogen-containing compound was added. Whenever there was a change in media, cultures were harvested by centrifugation (2,655×*g*, 15 min, 4°C), washed twice with phosphate buffer (5.4 g Na_2_HPO_4_·7H_2_O and 2.6 g KH_2_PO_4_ per litre distilled H_2_O) and finally resuspended in the desired medium. After each physiological experiment, purity of the culture was confirmed by fluorescence in situ hybridization (FISH) using a strain SC2-specific 16S rRNA-targeted oligonucleotide probe as described earlier [105] (Figure S3).

### N_2_ fixation assay

N_2_-fixing ability of strain SC2 was tested by batch incubation in N-free medium. Cells used for the assay were initially grown in NMS medium up to early logarithmic phase and washed properly to remove any residual nitrogen source. They were then inoculated in 20 ml N-free medium, resulting in an initial OD_600_ of 0.05. Incubation was done in 120-ml serum bottles that were sealed with butyl rubber stoppers. The bottles were flushed with N_2_. Methane (20% [vol/vol]) and the desired amount of oxygen (1, 5, 10, 15 or 20% [vol/vol]) were then injected into the headspace.

The acetylene reduction assay is widely used to test for nitrogenase activity in bacteria and was performed accordingly [21], [24], [90], [113]. To induce enzyme activity, cells were initially grown in N-free medium. Briefly, 5 ml of a suspension of log-phase cells (0.48 mg dry weight) were transferred to 25-ml serum bottles and sealed with butyl rubber stoppers. The bottles were flushed with N_2_-free helium gas. Oxygen in the headspace was then set to 1, 5, 10, 15, or 20% (vol/vol), as mentioned above. Methanol was added to a final concentration of 0.1% (vol/vol). Acetylene (10% [vol/vol]), which had been purified by successive passage through 2 M sulfuric acid and double-distilled water, was then injected. To measure the ethylene production, 0.5 ml of the gas phase was sampled at fixed time intervals and analyzed using a gas chromatograph. The gas chromatograph (GC 14b; Shimadzu, Griesheim, Germany) was equipped with a stainless steel column filled with Porapak R and a flame ionization detector. N_2_ was used as the carrier gas. Pure acetylene and ethylene were used for calibration and as standards. All gas chromatography systems were routinely calibrated with certified gas standards (Air Liquide GmbH, Kassel, Germany). In all measurements, signals were processed and chromatograms were integrated using the Peak Simple software (version 2.66, SRI Instruments, Torrence, CA, USA).

### Denitrification assay

The acetylene inhibition method [93], [94], [95] was used to verify the production of N_2_O by strain SC2. Briefly, NMS-grown log-phase cells (3.4 mg dry weight) were resuspended in 5 ml of fresh NMS medium supplemented with methanol (0.1% [vol/vol]) or without a carbon source in 25-ml serum bottles that were capped with butyl rubber stoppers. To make the system anaerobic, the headspace was flushed with N_2_ for 10 min. If aerobic conditions should be maintained, oxygen (as described above) was injected into the headspace. When needed, purified acetylene was added to inhibit the conversion of N_2_O to N_2_. The bottles were then incubated on a rotary shaker at 30°C and periodically analyzed for N_2_O in the headspace using a gas chromatograph (Carlo Erba Instruments, GC 8000) connected to a ^63^Ni-electron capture detector (ECD) [95]. Potential rates of N_2_O production were calculated by linear regression after correcting for N_2_O dissolved in the liquid phase using the Bunsen coefficient for N_2_O [114].

Using ^15^N-nitrate (K^15^NO_3_), a tracer experiment was performed to check denitrification-mediated formation of N_2_. Strain SC2 cells (3.5 mg dry weight) that were pre-grown in NMS up to log phase were washed twice and resuspended in 5 ml of fresh NMS medium containing K^15^NO_3_ (isotopic purity of 98% ^15^N; Sigma-Aldrich) as the only nitrogen source, in 25-ml serum bottles. A control set was also installed where K^15^NO_3_ was replaced with KNO_3_. The serum bottles were sealed tightly with butyl rubber stoppers and, to make the system anaerobic, flushed with N_2_-free helium for 10 min. Aerobic conditions were maintained as described above. Bottles were incubated at 30°C on a rotary shaker. The production of N_2_ and the isotopic composition of N_2_ in the headspace was analyzed with a GC-IRMS system [114]. As K^15^NO_3_ was the only nitrogen source, the masses 28 (^28^N_2_ [^14^N^14^N]) and 29 (^29^N_2_ [^14^N^15^N]) were ignored and the increase in mass 30 (^30^N_2_ [^15^N^15^N]) with time was used as a proof of denitrification [114], [115]. ^30^N_2_ production was determined after fifteen days of incubation, because production was below the detection limit during the initial days of incubation.

### Comparative genome analysis

Annotations of the chromosome and plasmid sequences of strain SC2 were performed using the Silver genome annotation interface (http://www.micro-genomes.mpg.de/). All CDS mentioned in the text have an E-value of 10^−10^ as cutoff.

Eight methanotroph genomes (Table 1) were used to setup a new comparative genomics project in the EDGAR server of the Center for Biotechnology, Bielefeld University, Bielefeld, Germany (http://edgar.cebitec.uni-bielefeld.de) [100]. For strain SC2 and *Mm. alcaliphilum* 20Z, concatenated sequences of their chromosome and the plasmid(s) were used. The strain SC2 genome was used as the reference in all comparative analyses. The EDGAR platform calculates so-called BLASTP score ratio values (SRV) and then defines orthologous proteins based on bidirectional best BLAST hits. As the genomes used in this comparative study represent a set of phylogenetically diverse bacteria, a comparably low SRV cutoff of 35 was used. As a consequence, paralogous genes might have been discarded during the analysis.

### Construction of a core genome tree

EDGAR was used to construct a phylogenetic tree based on 154 CDS common to all analyzed species (orthology-cutoff 35% SRV) [100]. The genomic sequences that were initially aligned sum up to 1,232 CDS with a total of 473,457 amino acids. Alignments of these core genes were generated using MUSCLE [116], with non-matching parts being masked by GBLOCKS and subsequently removed [117]. The remaining parts of all the individual gene alignments were compiled in one concatenated alignment. Pairwise distances between the concatenated core genome sequences were calculated using Kimura's two-parameter method. The distance matrix was used as input to construct a phylogenetic tree with the neighbor-joining method (implemented in the PHYLIP package). The final tree was created in Newick format and visualized in iTOL, a web server for visualizing phylogenetic trees (http://itol.embl.de/index.shtml).

### Classification of CDS into functional groups

To classify CDS present in a particular genome or a selected gene set (like the core genome) into functional groups, we used the RAST server (http://rast.nmpdr.org/rast.cgi). To achieve this classification, the gene sets (in GenBank format) were subjected to automated annotation process in the SEED subsystem using RAST, and gene calls were preserved as in the uploaded file [118]. This resulted in an output where the CDS were functionally classified into 27 distinct hierarchical categories. Analysis of significant differences in the number of CDS classified for the individual strains and their core genome into each SEED subsystem was performed using STAMP (Statistical Analysis of Metagenomic Profiles) [119].

## Supporting Information

Figure S1 **Venn diagrams showing the number of CDS unique to and shared by different methanotrophs.** Numbers in circles indicate the total number of CDS unique to each member, while those in intersections represent the number of orthologous CDS common to two or more methanotrophs. (A, B) Comparative genomics was performed between (A) four alphaproteobacterial methanotrophs [(1) *Methylocystis* sp. strain SC2, (2) *Mce. silvestris* BL2, (3) *Methylocystis* sp. strain Rockwell, and (4) *Ms. trichosporium* OB3b] and (B) three gammaproteobacterial methanotrophs [(1) *Mm. alcaliphilum* 20Z, (2) *Mc. capsulatus* Bath, and (3) *Mmo. methanica* MC09]. Orthologs were detected by reciprocal best BLASTP matches with the EDGAR software.
(TIF)

Figure S2 **Strain-specific differences in the number of CDS present in particular SEED subsystems relative to the core genome.** The number of CDS classified for the individual strains and their core genome into each SEED subsystem was subjected to statistical analysis using STAMP. A *p*-value cutoff of 0.05 was used for determining significant differences. Subsystems showing significant differences in strains SC2 (A), Rockwell (B) and OB3b (C) (blue), when compared to their core genome (orange), are shown.
(TIF)

Figure S3 **Purity check of strain SC2 by FISH.** Representative field of view showing cells of strain SC2: (A) phase contrast microscopy; (B, C) whole-cell hybridization with bacterial probe EUB338 (green) and species-specific probe Mcyst-1256 (red); (D) staining with DAPI (blue). Bar represents 10 µm.
(TIF)

Table S1 **Gene products that are known or likely to be involved in methane oxidation and nitrogen metabolism of** ***Methylocystis*** **sp. strain SC2.** Gene homologs identified in the draft genomes of strain Rockwell and *Ms. trichosporium* OB3b are shown in the last two columns by their respective locus tags.
(DOCX)

Table S2 **BLAST hits of the strain SC2 plasmid-encoded** ***nos*** **genes.**
(DOCX)

Table S3 **List of 154 CDS that form the core genome of the eight methanotrophs compared in this study and were used for the construction of the genome tree.**
(XLSX)
