# Supplementary figures and images for: Genome Analysis Coupled with Physiological Studies Reveals a Diverse Nitrogen Metabolism in Methylocystis sp. Strain SC2
Source: PLoS One. 2013 Oct 10;8(10):e74767. doi: 10.1371/journal.pone.0074767 (PMC3794950; doi:10.1371/journal.pone.0074767)

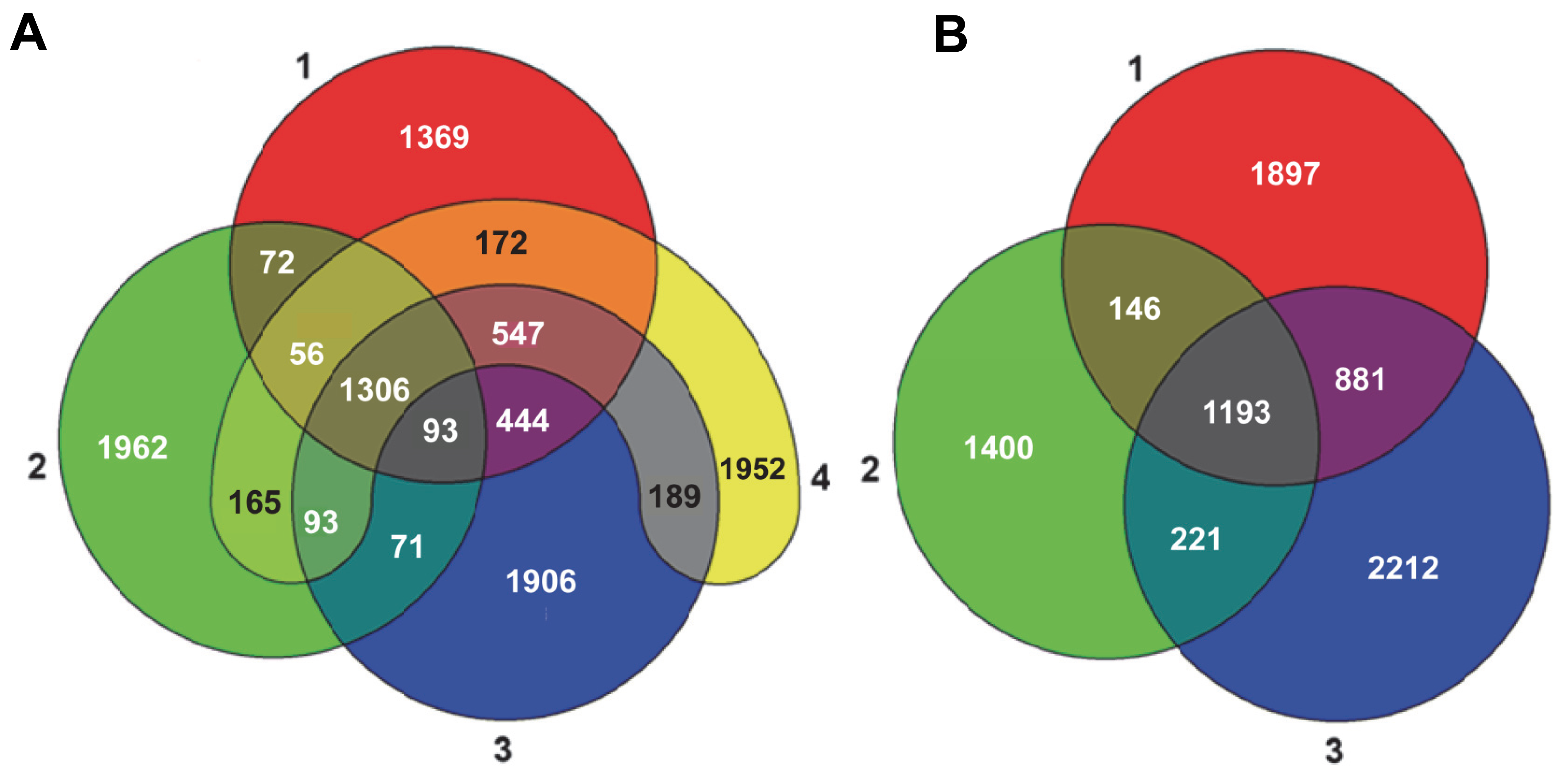

Supplement: Figure S1 — Venn diagrams showing the number of CDS unique to and shared by different methanotrophs. Numbers in circles indicate the total number of CDS unique to each member, while those in intersections represent the number of orthologous CDS common to two or more methanotrophs. (A, B) Comparative genomics was performed between (A) four alphaproteobacterial methanotrophs [(1) Methylocystis sp. strain SC2, (2) Mce. silvestris BL2, (3) Methylocystis sp. strain Rockwell, and (4) Ms. trichosporium OB3b] and (B) three gammaproteobacterial methanotrophs [(1) Mm. alcaliphilum 20Z, (2) Mc. capsulatus Bath, and (3) Mmo. methanica MC09]. Orthologs were detected by reciprocal best BLASTP matches with the EDGAR software. (TIF) [file pone.0074767.s001.tif]

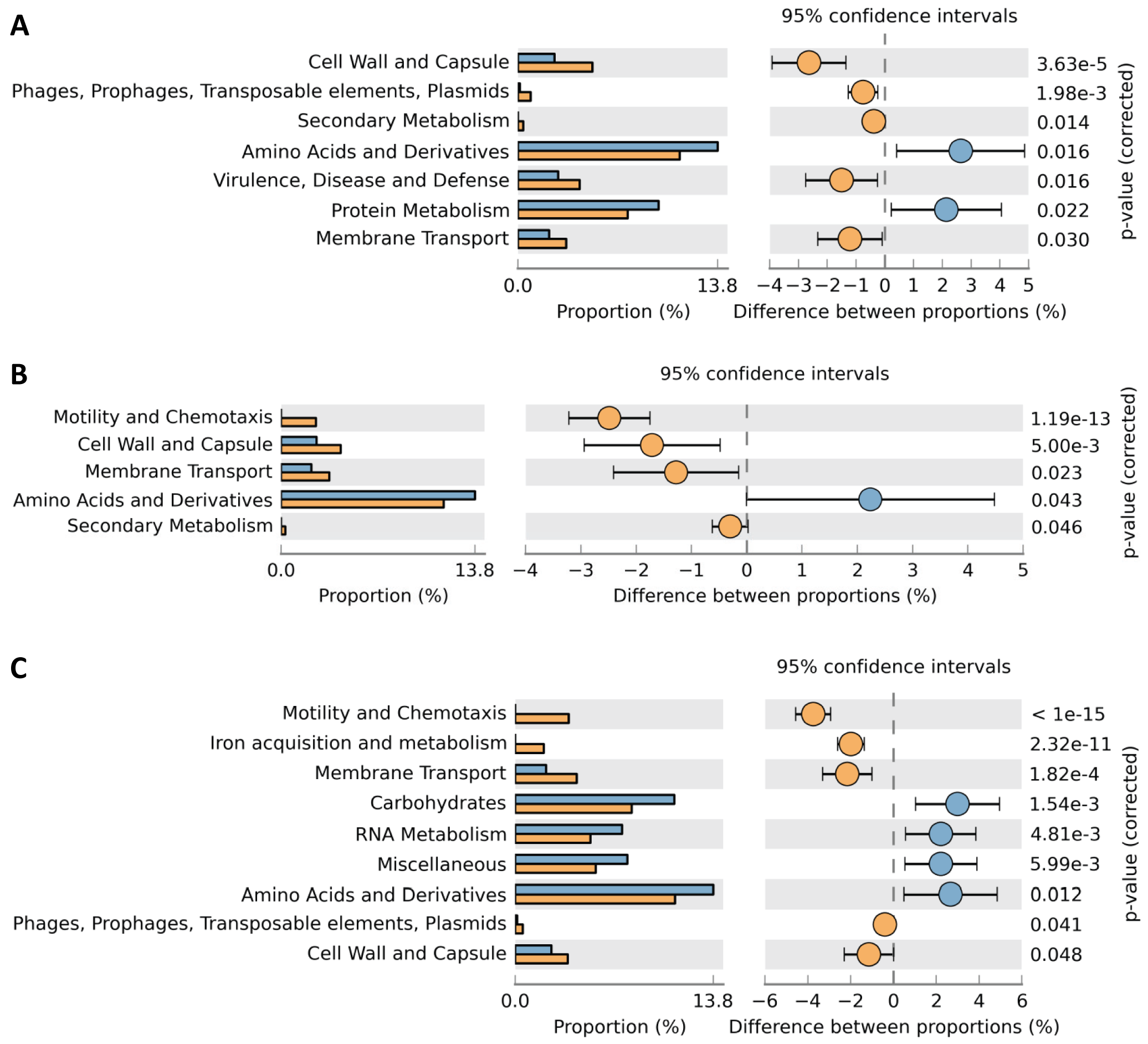

Supplement: Figure S2 — Strain-specific differences in the number of CDS present in particular SEED subsystems relative to the core genome. The number of CDS classified for the individual strains and their core genome into each SEED subsystem was subjected to statistical analysis using STAMP. A p-value cutoff of 0.05 was used for determining significant differences. Subsystems showing significant differences in strains SC2 (A), Rockwell (B) and OB3b (C) (blue), when compared to their core genome (orange), are shown. (TIF) [file pone.0074767.s002.tif]

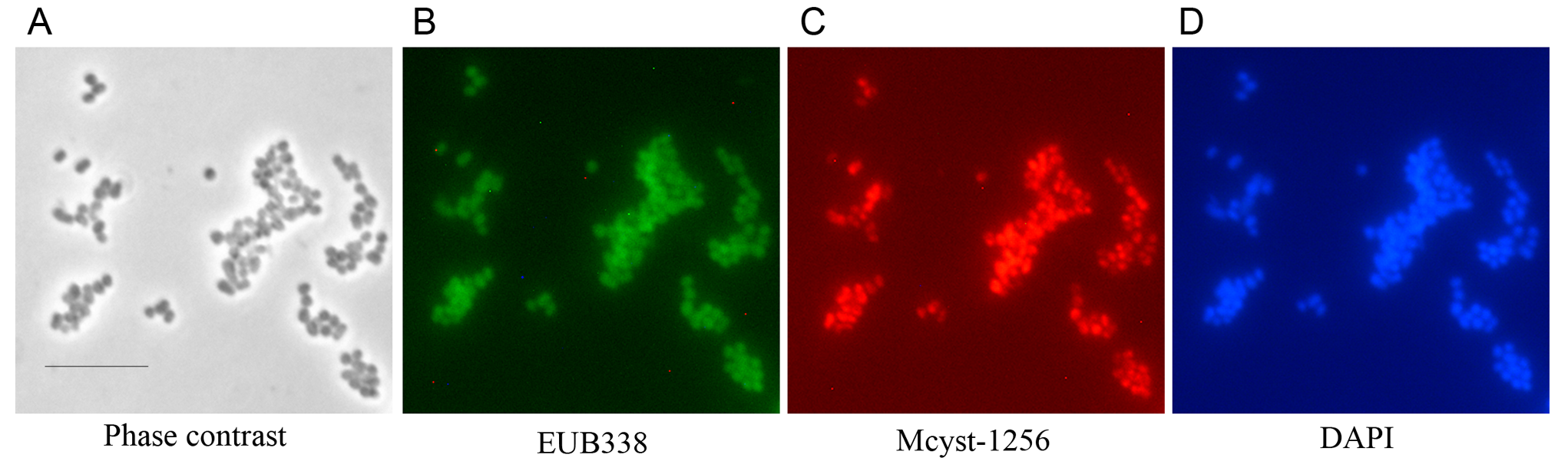

Supplement: Figure S3 — Purity check of strain SC2 by FISH. Representative field of view showing cells of strain SC2: (A) phase contrast microscopy; (B, C) whole-cell hybridization with bacterial probe EUB338 (green) and species-specific probe Mcyst-1256 (red); (D) staining with DAPI (blue). Bar represents 10 µm. (TIF) [file pone.0074767.s003.tif]
