# Supplementary material for: Genome Analysis Coupled with Physiological Studies Reveals a Diverse Nitrogen Metabolism in Methylocystis sp. Strain SC2
Source: PLoS One. 2013 Oct 10;8(10):e74767. doi: 10.1371/journal.pone.0074767 (PMC3794950; doi:10.1371/journal.pone.0074767)
Supplement: Table S1 — Gene products that are known or likely to be involved in methane oxidation and nitrogen metabolism of Methylocystis sp. strain SC2. Gene homologs identified in the draft genomes of strain Rockwell and Ms. trichosporium OB3b are shown in the last two columns by their respective locus tags. (DOCX) [file pone.0074767.s004.docx]

**Table S1. Gene products that are known or likely to be involved in methane oxidation and nitrogen metabolism of *Methylocystis* sp. strain SC2.** Gene homologs identified in the draft genomes of strain Rockwell and *Ms*. *trichosporium* OB3b are shown in the last two columns by their respective locus tags^1^.

| **Protein/ function** | **Gene name** | **E-value** | **Locus tag in strain SC2 chromosome/plasmids** | **Locus tag of homologs identified in the draft genome of *Methylocystis* sp. strain Rockwell** | **Locus tag of homologs identified in the draft genome of *Ms. trichosporium* OB3b** |
| --- | --- | --- | --- | --- | --- |
| ***Methane oxidation*** |  |  |  |  |  |
| Particulate methane monooxygenase subunit C1_Gs_ | *pmoC2_Gs_* | 9e-94 | BN69_0727 | Met49242DRAFT_3015 | MettrDRAFT_4397 |
| Particulate methane monooxygenase subunit C2_Gs_ | *pmoC1_Gs_* | 1e-120 | BN69_0852 | ND^2^ | ND |
| Particulate methane monooxygenase subunit C1 | *pmoC1_a_* | 1e-120 | BN69_2826 | Met49242DRAFT_3015 | MettrDRAFT_4397 |
| Particulate methane monooxygenase subunit C1 | *pmoC1_b_* | 1e-120 | BN69_3533 | Met49242DRAFT_3015 | MettrDRAFT_4397 |
| Particulate methane monooxygenase subunit C3_Ps_ | *pmoC3_Ps_* | 1e-109 | SC2p2_00560 | ND | MettrDRAFT_2626 |
| Particulate methane monooxygenase subunit A1 | *pmoA* | 1e-119 | BN69_282 | Met49242DRAFT_0659 | MettrDRAFT_4396 |
| Particulate methane monooxygenase subunit A1 | *pmoA* | 1e-119 | BN69_3534 | Met49242DRAFT_0659 | MettrDRAFT_4396 |
| Particulate methane monooxygenase subunit B1 | *pmoB* | 0 | BN69_2828 | Met49242DRAFT_0660 | MettrDRAFT_4395 |
| Particulate methane monooxygenase subunit B1 | *pmoB* | 0 | BN69_3535 | Met49242DRAFT_0660 | MettrDRAFT_4395 |
| Particulate methane monooxygenase subunit C2 | *pmoC2* | 1e-137 | BN69_0202 | ND | ND |
| Particulate methane monooxygenase subunit A2 | *pmoA2* | 1e-121 | BN69_0203 | ND | ND |
| Particulate methane monooxygenase subunit B2 | *pmoB2* | 0 | BN69_0204 | ND | ND |
| ***Methanol oxidation*** |  |  |  |  |  |
| Methanol dehydrogenase large subunit | *mxaF* | 0 | BN69_2570 | Met49242DRAFT_3163 | MettrDRAFT**_**2205 |
| MxaJ protein involved in methanol dehydrogenase | *mxaJ* | 1e-122 | BN69_2571 | Met49242DRAFT_3162 | MettrDRAFT**_**2204 |
| MxaG, cytochrome c class I, involved in methanol dehydrogenase | *mxaG* | 3e-79 | BN69_2572 | Met49242DRAFT_3161 | MettrDRAFT**_**2203 |
| Methanol dehydrogenase small beta subunit | *mxaI* | 4e-39 | BN69_2573 | Met49242DRAFT_3160 | MettrDRAFT_2202 |
| MxaR involved in methanol oxidation | *mxaR* | 1e-152 | BN69_2574 | Met49242DRAFT**_**3159 | MettrDRAFT_2201 |
| MxaS protein, involved in methanol oxidation | *mxaS* | 4e-84 | BN69_2575 | Met49242DRAFT**_**3158 | MettrDRAFT_2200 |
| MxaA protein involved in Ca^2+^ insertion into methanol dehydrogenase | *mxaA* | 8e-70 | BN69_2576 | Met49242DRAFT_3157 | MettrDRAFT_2199 |
| MxaC protein involved in Ca^2+^ insertion into methanol dehydrogenase | *mxaC* | 1e-111 | BN69_2577 | Met49242DRAFT_3156 | MettrDRAFT_2198 |
| MxaK protein involved in Ca^2+^ insertion into methanol dehydrogenase | *mxaK* | 1e-57 | BN69_2578 | Met49242DRAFT_3155 | MettrDRAFT_2197 |
| MxaL protein involved in Ca^2+^ insertion into methanol dehydrogenase | *mxaL* | 1e-114 | BN69_2579 | Met49242DRAFT_3154 | MettrDRAFT_2196 |
| MxaD-like protein | *mxaD* | 1e-68 | BN69_2580 | Met49242DRAFT_3153 | MettrDRAFT_2195 |
| Putative MxaH-like protein | *mxaH* | 4e-41 | BN69_2581 | Met49242DRAFT_3152 | MettrDRAFT_2194 |
| Methanol dehydrogenase-like protein XoxF | *xoxF* | 0 | BN69_1363 | Met49242DRAFT_0139 | MettrDRAFT_2210 |
| Putative dehydrogenase, XoxF precursor | *xoxF* | 2e-77 | BN69_3035 | Met49242DRAFT_2791 | MettrDRAFT_2212 |
| PQQ-dependent dehydrogenase, XoxF precursor | *-* | 1e-72 | BN69_3036 | Met49242DRAFT_0139 | MettrDRAFT_2210 |
| PQQ-dependent dehydrogenase, XoxF precursor | *-* | 1e-127 | BN69_3037 | Met49242DRAFT_0139 | MettrDRAFT_2210 |
| Putative methanol oxidation protein XoxJ | *xoxJ* | 1e-131 | BN69_1361 | Met49242DRAFT_0137 | MettrDRAFT_2212 |
| Putative methanol oxidation protein XoxG | *xoxG* | 3e-78 | BN69_1362 | Met49242DRAFT_0138 | MettrDRAFT_2211 |
| ***Pyrroloquinoline quinone (PQQ) biosynthesis*** |  |  |  |  |  |
| Pyrroloquinoline quinone biosynthesis protein B | *pqqB* | 1e-139 | BN69_3489 | Met49242DRAFT_2735 | MettrDRAFT_1619 |
| Pyrroloquinoline quinone biosynthesis protein C | *pqqC* | 1e-123 | BN69_3488 | Met49242DRAFT_2734 | MettrDRAFT_1618 |
| Pyrroloquinoline quinone biosynthesis protein E | *pqqE* | 0 | BN69_3487 | Met49242DRAFT_2733 | MettrDRAFT_1617 |
| ***Formaldehyde oxidation*** |  |  |  |  |  |
| NAD-dependent formate dehydrogenase delta subunit protein | *-* | 6e-21 | BN69_1448 | Met49242DRAFT_3923 | MettrDRAFT_2889 |
| Formate dehydrogenase family accessory protein FdhD | *-* | 1e-120 | BN69_1449 | Met49242DRAFT_3921 | MettrDRAFT_2888 |
| NAD-dependent formate dehydrogenase, alpha subunit | *-* | 0 | BN69_1450 | Met49242DRAFT_3920 | MettrDRAFT_3695 |
| ***Nitrogen fixation*** |  |  |  |  |  |
| Nif-specific regulatory protein | *nifA* | 1e-175 | BN69_2611 | Met49242DRAFT_2957 | MettrDRAFT_0294 |
| 4Fe-4S ferredoxin, nitrogenase-associated protein | *-* | 6e-18 | BN69_2612 | Met49242DRAFT_2955 | MettrDRAFT_1312 |
| LRV FeS4 cluster domain protein | *-* | 8e-46 | BN69_2613 | Met49242DRAFT_2952 | MettrDRAFT_0300 |
| FeS assembly protein SufB | *-* | 0 | BN69_2614 | Met49242DRAFT_0763 | MettrDRAFT_1504 |
| FeS assembly ATPase SufC | *-* | 1e-105 | BN69_2615 | Met49242DRAFT_0764 | MettrDRAFT_1505 |
| FeS assembly protein SufD | *-* | 3e-96 | BN69_2616 | Met49242DRAFT_0765 | MettrDRAFT_1506 |
| Nitrogenase FeMo cofactor biosynthesis protein NifB | *nifB* | 0 | BN69_2617 | Met49242DRAFT_2956 | MettrDRAFT_0296 |
| Ferredoxin-like protein | *-* | 8e-23 | BN69_2618 | Met49242DRAFT_2955 | MettrDRAFT_0297 |
| Putative iron-sulfur cluster assembly protein | *-* | 5e-26 | BN69_2619 | Met49242DRAFT_2954 | MettrDRAFT_0298 |
| Conserved Protein | *-* | 2e-51 | BN69_2620 | Met49242DRAFT_2953 | MettrDRAFT_0299 |
| LRV FeS cluster domain protein | *-* | 7e-75 | BN69_2621 | Met49242DRAFT_2952 | MettrDRAFT_0300 |
| Nitrogenase iron protein 2, NifH | *nifH* | 1e-156 | BN69_2623 | Met49242DRAFT_2940 | MettrDRAFT_0311 |
| Nitrogenase protein alpha chain, NifD | *nifD* | 0 | BN69_2624 | Met49242DRAFT_2939 | MettrDRAFT_0312 |
| Nitrogenase molybdenum-iron protein beta chain, NifD | *nifD* | 0 | BN69_2625 | Met49242DRAFT_2938 | MettrDRAFT_0313 |
| Nitrogenase MoFe cofactor biosynthesis protein NifE | *nifE* | 0 | BN69_2626 | Met49242DRAFT_2937 | MettrDRAFT_0314 |
| Nitrogenase molybdenum-iron cofactor biosynthesis protein NifN | *nifN* | 0 | BN69_2627 | Met49242DRAFT_2936 | MettrDRAFT_0315 |
| Nitrogen fixation protein NifX | *nifX* | 1e-58 | BN69_2628 | Met49242DRAFT_2935 | MettrDRAFT_0316 |
| NifX-associated protein | *-* | 7e-67 | BN69_2629 | Met49242DRAFT_2934 | MettrDRAFT_0317 |
| NifX-associated protein | *-* | 4e-11 | BN69_2630 | Met49242DRAFT_2933 | MettrDRAFT_0318 |
| Putative NifU protein | *-* | 3e-25 | BN69_2640 | Met49242DRAFT_2929 | MettrDRAFT_0322 |
| Nitrogenase metalloclusters biosynthesis protein NifS | *nifS* | 1e-146 | BN69_2641 | Met49242DRAFT_2928 | MettrDRAFT_0323 |
| Nitrogen fixation protein FixU | *fixU* | 1e-25 | BN69_2642 | Met49242DRAFT_2949 | MettrDRAFT_0303 |
| NifZ family protein | *-* | 2e-39 | BN69_2644 | Met49242DRAFT_2951 | MettrDRAFT_0301 |
| NifZ family protein | *-* | 1e-25 | BN69_2645 | Met49242DRAFT_2950 | MettrDRAFT_0302 |
| Nitrogenase iron protein 2, NifH | *nifH* | 1e-156 | BN69_2665 | Met49242DRAFT_2940 | MettrDRAFT_0311 |
| Putative nitrogen fixation protein NifQ | *nifQ* | 3e-60 | BN69_2666 | Met49242DRAFT_2931 | MettrDRAFT_0320 |
| NifU-like iron-sulfur cluster assembly protein | *-* | 6e-22 | BN69_2667 | Met49242DRAFT_2930 | MettrDRAFT_0321 |
| NifV protein, encodes a homocitrate synthase | *nifV* | 1e-123 | BN69_2668 | Met49242DRAFT_2927 | MettrDRAFT_0324 |
| NifP protein, serine O-acetyltransferase | *-* | 6e-85 | BN69_2669 | Met49242DRAFT_2926 | MettrDRAFT_0323 |
| Nitrogenase-stabilizing/protective protein NifW | *nifW* | 4e-28 | BN69_2670 | Met49242DRAFT_2925 | MettrDRAFT_0324 |
| FixA,electron transfer flavoprotein beta-subunit | *fixA* | 1e-116 | BN69_2671 | Met49242DRAFT_2924 | MettrDRAFT_0325 |
| FixB, electron transfer flavoprotein, alpha subunit | *fixB* | 1e-177 | BN69_2672 | Met49242DRAFT_2923 | MettrDRAFT_0328 |
| FixC protein, flavoprotein-ubiquinone oxidoreductase | *fixC* | 0 | BN69_2673 | Met49242DRAFT_2922 | MettrDRAFT_0329 |
| Putative ferredoxin protein, FixX | *fixX* | 1e-43 | BN69_2674 | Met49242DRAFT_2921 | MettrDRAFT_0330 |
| ***Nitrate/nitrite assimilation*** |  |  |  |  |  |
| Nitrate transporter component, NrtA | *-* | 1e-136 | BN69_2468 | Met49242DRAFT_3885 | MettrDRAFT_2605 |
| Nitrite reductase [NAD(P)H], large subunit | *nasD* | 1e-107 | BN69_2469 | Met49242DRAFT_3690 | MettrDRAFT_2606 |
| NAD(P)H-dependent nitrite reductase catalytic subunit | *nirA* | 0 | BN69_2470 | ND | MettrDRAFT_1827 |
| NAD(P)H-dependent nitrite reductase flavoprotein subunit | *-* | 0 | BN69_2471 | ND | ND |
| Nitrate reductase, large subunit | *nasA* | 0 | BN69_2472 | Met49242DRAFT_3692 | MettrDRAFT_2608 |
| Nitrate transporter | *-* | 1e-152 | BN69_2473 | Met49242DRAFT_3693 | MettrDRAFT_2609 |
| ***Ammonium transporter*** |  |  |  |  |  |
| Putative ammonium transporter | *-* | 0 | BN69_0915 | Met49242DRAFT_2997 | MettrDRAFT_1434 |
| Putative ammonium transporter | *-* | 0 | BN69_0931 | Met49242DRAFT_2240 | ND |
| ***Ammonia assimilation*** |  |  |  |  |  |
| Glutamine synthetase (GS) | *-* | 0 | BN69_0652 | Met49242DRAFT_2337 | MettrDRAFT_1973 |
| Glutamate synthetase (GOGAT), large subunit | *-* | 0 | BN69_3582 | Met49242DRAFT_0707 | MettrDRAFT_2587 |
| Glutamate synthetase (GOGAT), small subunit | *-* | 0 | BN69_3584 | Met49242DRAFT_0709 | MettrDRAFT_2563 |
| Glutamate dehydrogenase (GDH) | *-* | 1e-147 | BN69_0999 | Met49242DRAFT_1541 | MettrDRAFT_1875 |
| ***Nitrogen metabolism-related regulatory components*** |  |  |  |  |  |
| RNA polymerase sigma factor RpoN | *rpoN* | 0 | BN69_2202 | Met49242DRAFT_0327 | MettrDRAFT_0576 |
| tRNA-dihydrouridine synthase | *nifR3* | 3e-94 | BN69_0221 | Met49242DRAFT_0896 | MettrDRAFT_3010 |
| Signal transduction histidine kinase, nitrogen specific, NtrB | *ntrB* | 1e-178 | BN69_0222 | Met49242DRAFT_0897 | MettrDRAFT_3011 |
| Nitrogen metabolism transcriptional regulator, NtrC, Fis Family | *ntrC* | 0 | BN69_0223 | Met49242DRAFT_0898 | MettrDRAFT_3012 |
| Multi-sensor signal transduction histidine kinase, NtrY | *ntrY* | 1e-153 | BN69_0224 | Met49242DRAFT_0900 | MettrDRAFT_3015 |
| Nitrogen assimilation regulatory protein, NtrX | *ntrX* | 0 | BN69_0225 | Met49242DRAFT_0901 | MettrDRAFT_3016 |
| ***Nitrification/ hydroxylamine detoxification*** |  |  |  |  |  |
| Hydroxylamine oxidoreductase subunit | *hoaA* | 0 | BN69_3242 | Met49242DRAFT_3480 | ND |
| Hydroxylamine oxidoreductase subunit | *hoaB* | 1e-106 | BN69_3241 | Met49242DRAFT_3479 | ND |
| Hydroxylamine reductase | *hcp* | 0 | BN69_0431 | ND | MettrDRAFT_2184 |
| Hydroxylamine reductase (N-terminal region)^3^ | *hcp* | 7e-98 | SC2p2_01470 | ND | MettrDRAFT_2184 |
| Hydroxylamine reductase (C-terminal region)^3^ | *hcp* | 1e-119 | SC2p2_01480 | ND | MettrDRAFT_2184 |
| ***Denitrification*** |  |  |  |  |  |
| NorB/NorZ-like protein, nitric-oxide reductase | *norB/norZ* | 0 | SC2p1_00450 | ND | ND |
| NorB/NorZ-like protein, nitric-oxide reductase | *norB/norZ* | 0 | SC2p2_01300 | ND | ND |
| ApbE family lipoprotein | *nosX* | 3e-72 | SC2p2_01390 | ND | ND |
| Nitrous-oxide reductase | *nosY* | 4e-60 | SC2p2_01400 | ND | ND |
| copper ABC transporter ATP-binding protein | *nosF* | 3e-75 | SC2p2_01410 | ND | ND |
| Periplasmic copper-binding protein | *nosD* | 1e-128 | SC2p2_01420 | ND | ND |
| Nitrous oxide reductase | *nosZ* | 0 | SC2p2_01430 | ND | ND |
| Nitrous oxide reductase expression regulator | *nosR* | 0 | SC2p2_01440 | ND | ND |
| NnrS protein (probable denitrification associated gene) | *-* | 9e-72 | SC2p1_00430 | ND | ND |
| NnrU family protein (probable denitrification associated gene) | *-* | 1e-69 | BN69_1205 | Met49242DRAFT_3364 | ND |

^1^Gene homologs were identified by BLAST search of the individual strain SC2 genes against the draft genome sequences of strain Rockwell and *Ms*. *trichosporium* OB3b.

^2^ND, Not detected in the respective genome sequence.

^3^The two subunits probably encode a single protein in the cell. This artifact is probably due to some sequencing errors.
