# Supplementary material for: Genome Analysis Coupled with Physiological Studies Reveals a Diverse Nitrogen Metabolism in Methylocystis sp. Strain SC2
Source: PLoS One. 2013 Oct 10;8(10):e74767. doi: 10.1371/journal.pone.0074767 (PMC3794950; doi:10.1371/journal.pone.0074767)
Supplement: Table S2 — BLAST hits of the strain SC2 plasmid-encoded nos genes. (DOCX) [file pone.0074767.s005.docx]

**Table S2. BLAST hits of the strain SC2 plasmid-encoded *nos* genes.**

| **Gene ID** | **Gene** | **Size (aa)** | **Protein** | **Best BLAST hit with** | **E-value** | **Similarity (length aligned / total length of target protein)** |
| --- | --- | --- | --- | --- | --- | --- |
| SC2p2_01390 | *nosX* | 331 | ApbE family lipoprotein | *Afipia* sp. 1NLS2 | 3e-72 | 48% (322/331) |
| SC2p2_01400 | *nosY* | 276 | Nitrous oxide reductase | *Simonsiella muelleri* ATCC 29453 *Bradyrhizobium* sp. strain BTAi1 | 4e-60 1e-59 | 52% (273/276) 59% (275/276) |
| SC2p2_01410 | *nosF* | 307 | Copper ABC transporter ATP-binding protein | uncultured bacterium 1042 *Sinorhizobium meliloti* | 3e-75 6e-74 | 52% (301/307) 54% (296/304) |
| SC2p2_01420 | *nosD* | 462 | Periplasmic copper-binding protein | *Rhodospirillum centenum* strain ATCC 51521/SW | e-128 | 59% (402/432) |
| SC2p2_01430 | *nosZ* | 642 | Nitrous oxide reductase | *Rhodospirillum centenum* strain ATCC 51521/SW | 0.0 | 72% (632/642) |
| SC2p2_01440 | *nosR* | 730 | Nitrous oxide reductase expression regulator | *Azospirillum brasilense* *Rhodopseudomonas palustris* DX-1 | 0.0 0.0 | 57% (726/730) 54% (745/764) |
